# Generation of Recombinant Polioviruses Harboring RNA Affinity Tags in the 5′ and 3′ Noncoding Regions of Genomic RNAs

**DOI:** 10.3390/v8020039

**Published:** 2016-02-04

**Authors:** Dylan Flather, Andrea L. Cathcart, Casey Cruz, Eric Baggs, Tuan Ngo, Paul D. Gershon, Bert L. Semler

**Affiliations:** 1 Department of Microbiology and Molecular Genetics, School of Medicine, University of California, Irvine, CA 92697, USA; dflather@uci.edu (D.F.); andrea.cathcart@gilead.com (A.L.C.); cruzc3@uci.edu (C.C.); ebaggs@uci.edu (E.B.); 2 Present address: Gilead Sciences, Inc., Foster City, CA 94404, USA; 3 Department of Molecular Biology and Biochemistry, University of California, Irvine, CA 92697, USA; tngo5@uci.edu (T.N.); pgershon@uci.edu (P.D.G.)

**Keywords:** enterovirus RNA replication, RNA replication complexes, MS2 hairpin, aptamer, ribonucleoprotein complexes, RNP isolation, RNA affinity

## Abstract

Despite being intensely studied for more than 50 years, a complete understanding of the enterovirus replication cycle remains elusive. Specifically, only a handful of cellular proteins have been shown to be involved in the RNA replication cycle of these viruses. In an effort to isolate and identify additional cellular proteins that function in enteroviral RNA replication, we have generated multiple recombinant polioviruses containing RNA affinity tags within the 3′ or 5′ noncoding region of the genome. These recombinant viruses retained RNA affinity sequences within the genome while remaining viable and infectious over multiple passages in cell culture. Further characterization of these viruses demonstrated that viral protein production and growth kinetics were unchanged or only slightly altered relative to wild type poliovirus. However, attempts to isolate these genetically-tagged viral genomes from infected cells have been hindered by high levels of co-purification of nonspecific proteins and the limited matrix-binding efficiency of RNA affinity sequences. Regardless, these recombinant viruses represent a step toward more thorough characterization of enterovirus ribonucleoprotein complexes involved in RNA replication.

## 1. Introduction

The *Picornaviridae* family of viruses is a group of small, non-enveloped viruses that contain single-stranded, positive-polarity RNA genomes. Picornaviruses are significant pathogens of humans because they are widespread and capable of causing serious diseases such as poliomyelitis, hepatitis, meningitis, and encephalitis as well as less serious diseases including the common cold. The enteroviruses are a single genera within the picornavirus family, of which poliovirus is the type species. Due to the inherent limited protein coding capacity of their small RNA genomes, enteroviruses require the functions of cellular proteins to complete their infectious cycle. Because enteroviral replication is composed of a series of discrete steps that demand particular protein functions, there are dynamic changes to the composition of ribonucleoprotein (RNP) complexes throughout this cycle.

Much of what is known about the identity of cellular proteins that are usurped during the replication cycle of enteroviruses is a result of studies involving poliovirus. The poliovirus genome consists of a small viral protein (VPg) covalently linked to the RNA at the very 5′ terminus followed by a relatively long (742 nucleotide) and highly structured 5′ noncoding region (5′NCR). There are six stem-loop (S-L I-VI) structures within the 5′NCR, with the internal ribosome entry site (IRES) comprised of S-L II-VI. Downstream of the 5′NCR the poliovirus genome encodes a single open reading frame. The 3′ region of the genome contains the ~75 nucleotide 3′ noncoding region (3′NCR), made up of two predicted stem-loop structures called X and Y, and the genetically encoded poly(A) tract of ~60 nucleotides [1]. The function of the 3′NCR is not clear, but the poly(A) tract is required for infectivity and is the putative binding site for the viral RNA-dependent RNA polymerase (3D^pol^) during initiation of negative-sense RNA synthesis [2,3].

Following cellular entry and uncoating, the initial step in the replication cycle of poliovirus is the translation of the ~7500 nucleotide genomic RNA molecule in the cytoplasm of the infected cell. Unlike cellular mRNAs, the poliovirus genome lacks a 5′ 7-methylguanosine cap structure and relies on cap-independent, IRES-mediated translation resulting in the production of a single 250-kDa polyprotein. The polyprotein is proteolytically processed by viral proteinases to produce 11 mature proteins, as well as intermediate precursor proteins, which have distinct functions. In addition to generating the proteins required for viral RNA replication directly, translation of the viral genome also produces proteins that alter the infected cell to support conditions required for viral RNA synthesis. This includes induction of membranous structures that originate from the secretory pathway and/or autophagosomal pathways during infection [4,5,6,7,8,9,10]. Viral RNA is synthesized in close association with these membranous structures induced during infection, and are together known as replication complexes [11].

Once sufficient levels of viral proteins have been produced, the genomic RNA that was a template for translation is subsequently used as a template for the generation of viral RNA molecules. This involves a template usage switch that is dependent, in part, upon cleavage of the host-cell protein polypyrimidine tract-binding protein 1 (PTB1) and/or poly(rC)-binding protein 2 (PCBP2), by the viral proteinase 3CD [12,13]. Poliovirus RNA replication can be divided into two distinct stages: (i) the production of negative-sense RNA intermediates from genomic RNA templates and (ii) the synthesis of nascent genomic RNAs (positive-sense RNA molecules) from negative-sense templates. Due to the asymmetric nature of enterovirus RNA replication, the ratio of positive- to negative-sense RNAs within an infected cell is approximately 50:1 [14,15,16]. Consequently, the majority of viral RNA in infected cells is single-stranded and positive-sense. Poliovirus RNA replication is dependent upon the formation of ribonucleoprotein (RNP) complexes that result from interactions between structured regions of the viral RNA molecule and proteins of host and viral origin. The RNA structures that nucleate various RNP complexes are found in the positive-sense RNA within in the 5′ NCR, the *cis*-acting replication element (*cre*) (a hairpin structure found within the coding region of 2C involved in the initiation of RNA synthesis), and the 3′NCR. RNP complexes function in stimulating viral RNA replication, through direct or indirect recruitment of 3D^pol^, and are thought to be the determinants of 3D^pol^ template specificity.

The RNA structural element known as the cloverleaf or S-L I, located at the very 5′ terminus of poliovirus genomic RNA, is required for efficient synthesis of negative-sense viral RNA, by acting as a scaffold for protein associations [14,17,18]. Poliovirus proteinase/polymerase precursor 3CD and host protein PCBP2 interact with the S-L I structure forming a ternary complex on the 5′ terminus of the template [19,20,21]. On the opposite end of the same RNA molecule, poly(A)-binding protein 1 (PABP1) associates with the 3′ poly(A) tract. Because PABP1 has been shown to interact with both PCBP2 as well as 3CD, the current model of negative-sense RNA synthesis is based on the circularization of the genomic template through interaction of the proteins present in the ternary complex and PABP1 [22]. End-to-end interaction is predicted to bring 3D^pol^ (perhaps in the form of 3CD that is bound to S-L I) into close proximity to the site of replication initiation at the 3′ terminus of the template RNA. Many features of this model have been verified *in vitro*, and the specificity of 3D^pol^ for viral RNA templates containing these RNP complexes is bolstered by the requirement for translation in *cis*, *i.e.*, a viral RNA template must be translated before being used for RNA replication. As a result, RNA templates enter the replication cycle already associated with various cellular proteins that function in the translation of the viral genome, and may also be required for efficient RNA replication [23]. Because negative-sense RNA templates are not translated, they likely have a unique set of viral and host protein requirements compared to their genomic RNA counterparts. Previous studies have identified a cellular protein that promotes positive-sense RNA synthesis: heterogeneous nuclear ribonucleoprotein C1/C2 (hnRNP C1/C2). hnRNP C1/C2 interacts with RNA sequences that correspond to both the 5′ and 3′ terminal regions of negative-sense RNA intermediates (regions complementary to the 3′NCR and 5′NCR of genomic RNA, respectively) [24,25,26]. The association of hnRNP C1/C2 with both termini of the negative-sense RNA molecule has been proposed to promote the end-to-end interaction of this RNA species through multimerization of hnRNP C1/C2 isoforms [27]. Newly synthesized positive-sense RNA molecules are subsequently used as templates for translation, recycled back into the RNA replication cycle, or packaged into viral capsids producing progeny virions that are released from the cell.

Studies that have contributed to our understanding of enterovirus RNA replication have defined several proteins that interact directly with poliovirus RNA during the process of viral RNA production. However, previous work has relied heavily on *in vitro* assays and subgenomic poliovirus RNA constructs. To define the RNP complexes using full length genomic RNA in the context of infection, we have generated recombinant polioviruses containing RNA affinity tags within the noncoding regions of the genome. The use of polioviruses possessing stable, specifically isolatable genomes and replication intermediates could ultimately allow for strand-specific RNP complex characterization directly from infected cells, throughout the course of infection. Here we present a biological characterization of these recombinant viruses and demonstrate that exogenous sequence insertions can be stable in the poliovirus genome for multiple passages in cell culture while maintaining wild type-like growth kinetics. Our initial attempts to isolate these genetically-tagged viral RNAs and associated proteins from infected cells has been hampered by high levels of co-purification of nonspecific proteins and the limited binding efficiency between RNA affinity sequences and their respective matrices. Nonetheless, our results provide a foundation for the generation of enteroviruses that could eventually allow for a description of the dynamic changes in protein composition of viral RNP complexes that occur throughout the course of infection, and that reflect the distinct steps of the viral RNA replication cycle.

## 2. Materials and Methods

### 2.1. Cell Culture and DNA Constructs

HeLa cells were grown as monolayers in Dulbecco’s Modified Eagle’s Medium (DMEM) or in suspension culture in Spinner Minimal Essential Medium (S-MEM), both supplemented with 8% newborn calf serum (NCS). To generate plasmids encoding MS2 tags in place of the 3′NCR, a HpaI site corresponding to nucleotide position 7378 in pT7PV1 [28] was engineered into the pT7PV1(∆3′NCR) plasmid [29]. Oligonucleotides corresponding to tandem MS2 hairpins were inserted into pT7PV1(∆3′NCR)HpaI at the HpaI site using blunt end ligation to generate pT7PV1-3′MS2 plasmid (MS2 stem-loop sequence obtained from SP73-βglobin-(MS2)4, kindly provided by Klemens Hertel (University of California, Irvine, CA, USA)). To generate plasmids encoding either the S1 or D8 aptamer tags in forward or reverse orientation, or the modified forms of these aptamers, within the 5′NCR of the poliovirus genome, three separate vectors were used. For constructs containing aptamer tag in place of S-L VI, the X585R plasmid [30] was engineered to contain an XhoI site at nucleotide position 564 (X585RXhoI). For generating aptamer tags in place of S-L III, and at nucleotide position 702, pT7PV1 was digested with HincII and re-ligated to generate a subclone of this vector. XhoI sites were inserted flanking S-L III or at nucleotide position 702. These subclones, and X585RXhoI, were digested with XhoI then ligated with double stranded oligonucleotides corresponding to each one of the tag sequences containing XhoI recognition sites (Table 1). Tagged subclones were then ligated into full-length poliovirus cDNA constructs.

**Table 1:** Oligonucleotides corresponding to aptamer tag sequences incorporated into the 5′ noncoding region (5′NCR) of the poliovirus genome cDNA.

Affinity Tag	Oligonucleotide Sequence (5′–3′)
S1+	top: TCGAACCGACCAGAATCATGCAAGTGCGTAAGATAGTCGCGGGCCGGG bottom: TCGACCCGGCCCGCGACTATCTTACGCACTTGCATGATTCTGGTCGGT
S1−	top: TCGACCCGGCCCGCGACTATCTTACGCACTTGCATGATTCTGGTCGGT bottom: TCGAACCGACCAGAATCATGCAAGTGCGTAAGATAGTCGCGGGCCGGG
S1m+	top: TCGAAAGCGGCCGCCGACCAGAATCATGCAAGTGCGTAAGATAGTCGCGGGTCGGCGGCCGCTT bottom: TCGAAAGCGGCCGCCGACCCGCGACTATCTTACGCACTTGCATGATTCTGGTCGGCGGCCGCTT
S1m−	top: TCGAAAGCGGCCGCCGACCCGCGACTATCTTACGCACTTGCATGATTCTGGTCGGCGGCCGCTT bottom: TCGAAAGCGGCCGCCGACCAGAATCATGCAAGTGCGTAAGATAGTCGCGGGTCGGCGGCCGCTT
D8+	top: TCGATCCGAGTAATTTACGTTTTGATACGGTTGCGGA bottom: TCGATCCGCAACCGTATCAAAACGTAAATTACTCGGA
D8−	top: TCGATCCGCAACCGTATCAAAACGTAAATTACTCGGA bottom: TCGATCCGAGTAATTTACGTTTTGATACGGTTGCGGA
D8m+	top: TCGAAAGCGGCCTCCGAGTAATTTACGTTTTGATACGGTTGCGGAGGCCGCTT bottom: TCGAAAGCGGCCTCCGCAACCGTATCAAAACGTAAATTACTCGGAGGCCGCTT
D8m−	top: TCGAAAGCGGCCTCCGCAACCGTATCAAAACGTAAATTACTCGGAGGCCGCTT bottom: TCGAAAGCGGCCTCCGAGTAATTTACGTTTTGATACGGTTGCGGAGGCCGCTT

### 2.2. RNA Constructs and Virus Stocks

RNA corresponding to full-length PV1 harboring MS2 hairpins or aptamer tags within the 5′NCR was generated by *in vitro* transcription of plasmids linearized with EcoRI using the MEGAscript T7 transcription kit (Ambion, Waltham, MA). Following transcription, RNA was purified by phenol/chloroform extraction followed by two rounds of ethanol precipitation in the presence of 700 mM ammonium acetate or by using the RNeasy Mini kit (Qiagen, Hilden, Germany). For transfection of RNA into HeLa cells, 1 μg of transcribed RNA was incubated with TS buffer (137 mM NaCl, 4.4 mM KCl, 0.7 mM Na_2_HPO_4_, 0.5 mM MgCl_2_, 0.68 mM CaCl_2_, 25 mM Tris, pH 7.5) and 1 mg/mL DEAE-Dextran. Cells were washed twice with phosphate buffered saline (PBS) and 250 μL of Diethylaminoethyl(DEAE)-Dextran transfection mixture was added per 20 cm^2^ plate of HeLa cells. After 30 min incubation at room temperature, DMEM-8% NCS was added and cells were incubated at 37 °C and monitored for cytopathic effects (CPE). CPE was observed after 4 days for MS2-containing RNA transfections or after 1 day for RNAs containing aptamer tags in the 5′NCR. Following detection of CPE, cells and supernatant were collected, subjected to 3-5 freeze-thaw cycles, and used to infect HeLa cell monolayers with a semi-solid agar overlay (DMEM-6% NCS, 0.45% agarose). Single plaque isolates were recovered at 2 (aptamer-tagged RNA transfections) or 4 (MS2-tagged RNA transfections) days post-infection and used to infect fresh HeLa cell monolayers. The resulting virus was designated as passage 1. Virus was amplified by serial passage at a multiplicity of infection (MOI) of 20 in HeLa cell monolayers. Large-scale virus preparations of PV1-3’MS2 were generated for passages 3 and 4 using HeLa suspension cells (400 mL). Cells were pelleted, washed once with PBS, washed once with S-MEM, and infected at an MOI of 0.2 in 370 mL S-MEM. After 30 min adsorption at room temperature, NCS was added to 10% and infection was allowed to proceed at 37 °C for 11 h. Cells were pelleted at ~1500 relative centrifugal units (rcf), resuspended in 25 mL DMEM, and subjected to five freeze-thaw cycles to release virus. Large-scale preparations of D8+/D8−/S1+/S1−/D8m+/D8m−702PV1 were generated for passage 3 stocks using HeLa suspension cells (1 liter). Cells were pelleted, washed once with PBS, resuspended with S-MEM and infected at an MOI of 20. After 30 min adsorption at room temperature, NCS was added to 8% by volume and infection was allowed to proceed for 8 h. Cell cultures and fluids were subjected to three freeze-thaw cycles prior to centrifugation at ~1500 rcf. For single-cycle growth analysis, HeLa cell monolayers were washed twice with PBS and infected with wild type PV1, PV1-3′MS2, or D8+/D8−/S1+/S1−/D8m+/D8m−702PV1 at an MOI of 20 for 30 min at room temperature. Cells were washed twice in PBS, overlaid with DMEM-8% NCS, and incubated at 37 °C. At times indicated, cells and supernatant were collected, subjected to 3–5 freeze-thaw cycles to release virus, and virus yields were determined in HeLa cells by plaque assay.

### 2.3. RT-PCR Assays

For sequencing of viral RNA, HeLa cells were infected at an MOI of 5 for 6 h (PV1-3′MS2) or an MOI of 20 for 4 h (D8+/D8−/S1+/S1−/D8m+/D8m−702PV1) and total cellular RNA was extracted using TriReagent (Molecular Research Center, Inc., Cincinnati, OH, USA) or TRIzol (Invitrogen, Carlsbad, CA, USA). Reverse transcription was performed using avian myeloblastosis virus (AMV) reverse transcriptase (Life Sciences, Inc., St. Petersburg, FL, USA). PCR amplification was performed using PfuTurbo (Stratagene, San Diego, CA, USA) and PCR products were purified with QIAquick PCR purification kit (Qiagen) prior to sequencing (Laguna Scientific, Laguna Hills, CA, USA). RNA primers complementary to poliovirus positive-strand nucleotide 17 to 35, 771 to 798, 1534 to 1557, 2725 to 2742, 3510 to 3528, 4230 to 4248, 5719 to 5738, 6549 to 6572, and the reverse complements of each were used for PV1-3′MS2 RT-PCR and sequencing, while those listed in [31] were used for D8+/D8−/S1+/S1−/D8m+/D8m−702PV1 and wild type poliovirus. For detection of MS2 hairpins and sequencing of the last 850 nucleotides of the viral RNA, MS2dT (5′-TTTTTTTTTGTTGACATGGGT-3′) or dT primer (5′-(T)_20_-3′) and PV3CD6605 (5′-AAAAACCCAGGAGTGATAACAG-3′) primers were used. For detection of viral RNA after MS2 purification, 50% of purified RNA was used for reverse transcription (RT) reactions; 50% of the RT reaction was used in PCR amplification using previously described primers corresponding to the 3C coding region of poliovirus [27]. For sequencing the 5′NCR of D8+/D8−/S1+/S1−/D8m+/D8m−702PV1 viruses, PV17+ (5′-GTTGTACCCACCCCAGAGG-3′) and PV895− (5V-CCTTGATGGGCTCGGTGAACTTG-3′) primers were used for RT-PCR.

### 2.4. Immunofluorescence Assays

HeLa cells were seeded on coverslips then infected with PV1 or PV1-3′MS2 at an MOI of 20 and fixed with 3.7% formaldehyde for 15 min at indicated times post-infection. Cells were permeabilized with 0.5% NP-40 in PBS for 5 min, followed by three washes in 1% NCS-PBS. Cells were incubated with anti-3A monoclonal antibody (1:500) followed by Alexa Fluor 488 goat anti-rabbit IgG (Molecular Probes; 1:1000). To stain the nucleus, cells were incubated with 4′,6-diamidino-2-phenylindole (DAPI) for 10 min. Cells were washed after each incubation with 1% NCS-PBS. Proteins were visualized with a Zeiss LSM700 laser scanning confocal microscope and images were processed with Zen software (Zeiss, Germany). Poliovirus 3A antibody was generously provided by George Belov (University of Maryland, College Park, MD, USA).

### 2.5. Translation of Viral Proteins During Infection and in Vitro

To radiolabel viral translation products in infected cells, HeLa cell monolayers were infected with PV1 or PV1-3′MS2 at an MOI of 10. Following a 30 min adsorption, DMEM without methionine (MP)-8% NCS was added to cells. Cells were pulsed with 20 μCi ^35^S-methionine (PerkinElmer, Waltham, MA, USA) 1 h prior to collection. Cells were washed twice with PBS, harvested, and resuspended in 2X Laemmli sample buffer (LSB). Crude lysates (25% of total) were resolved on a 12.5% polyacrylamide, SDS-containing gel. For *in vitro* translation assays, HeLa cell S10 cytoplasmic extracts were generated as described elsewhere [32]. HeLa S10 (60% of total volume) was incubated with 250 or 500 ng of *in vitro* transcribed RNA constructs corresponding to aptamer-tagged viral genomes or poliovirus virion RNA (vRNA), ^35^S-methionine (PerkinElmer), and all-four buffer (1 mM ATP, 0.25 mM GTP, 0.25 mM UTP, 0.25 mM CTP, 60 mM potassium acetate, 30 mM creatine phosphate, 0.4 mg/mL creatine kinase, 15.5 mM HEPES-KOH [pH 7.4]). Translation was allowed to proceed at 30 °C for 5–6 h. 2X LSB was added to an equal volume of translation reaction and boiled for 3 min. Samples were then subjected to electrophoresis on an SDS-containing 12.5% polyacrylamide gel. Proteins were visualized by autoradiography following fluorography. Quantity One software (Bio-Rad Laboratories, Hercules, CA, USA) was used to quantify VP3 band intensity of *in vitro* translation reactions that contained 250 ng of RNA relative to the band intensity of the poliovirus vRNA translation.

### 2.6. Purification of Recombinant MBP-MS2 Coat Protein

*E. coli* expressing MBP-MS2 was a gift from Yongsheng Shi (University of California, Irvine, CA, USA). Cells were grown at 37 °C until OD600 reached 0.4, and protein expression was induced with 0.5 mM isopropyl β-d-thiogalactoside (IPTG). After 3 h incubation at 37 °C, cells were pelleted, resuspended in lysis buffer (20 mM Tris HCl, pH 7.5, 200 mM NaCl, 0.5 mM PMSF), and lysed by sonication on ice 3 times at 30-second intervals. Lysates were cleared by centrifugation at ~7500 rcf for 10 min at 4 °C. The supernatant was added to washed amylose resin (NEB). Samples were incubated overnight at 4 °C with micrococcal nuclease and 5 mM CaCl_2_. The amylose resin was washed 3 times with lysis buffer, and MBP-MS2 was eluted with 5 mM Na_2_PO_4_, pH 7. Eluted protein was added to washed heparin agarose (Sigma-Aldrich, St. Louis, MO, USA) and incubated overnight at 4 °C. The heparin agarose was washed once with 5 mM Na_2_PO_4_, pH 7 and protein was eluted with buffer D-10% glycerol (20 mM HEPES, 100 mM KCl, 1 mM MgCl_2_, 0.2 mM EDTA) for 15 min at 4 °C. Protein concentration was determined by Bradford assay (Bio-Rad Laboratories).

### 2.7. MBP-MS2 RNA Affinity Purification

To generate lysate for MBP-MS2 RNA affinity, HeLa cell monolayers were infected with PV1-3′MS2 or wild type PV1 at an MOI of 5. After 30 min adsorption at room temperature, DMEM-8% NCS was added and cells were incubated at 37 °C for 4 h (wild type PV1) or 4 to 6 h (PV1-3′MS2). At indicated times post-infection, cells were washed twice with warm PBS, followed by cross linking with 0.4% formaldehyde in PBS for 10 min at room temperature with shaking. Formaldehyde was quenched with 0.25 M glycine for 5 min at room temperature. Cells were washed twice with ice-cold PBS, scraped, and pelleted at ~1000 rcf for 5 min at 4 °C. Cells were lysed in 100 μL NP-40 lysis buffer (50 mM Tris-HCl, pH 7.5, 5 mM EDTA, 150 mM NaCl, 1% NP-40) per 100 cm^2^ plate of HeLa cells for 25 min on ice. Cell debris was pelleted, supernatant was collected, and protein concentration was determined via Bradford assay.

RNA affinity resin was generated by incubating 40 μg recombinant MBP-MS2 protein per 10 μL washed magnetic amylose resin (NEB) in buffer B (20 mM Tris HCl, pH 7.5, 100 mM KCl, 2.5 mM MgCl_2_, 2 mM DTT, 0.5 mM Pefabloc SC (Roche, Basel, Switzerland), 20 U/mL RNasin (Promega, Madison, WI, USA)) for 1 h with rotation at 4 °C. Unbound MBP-MS2 was removed, and the resin was blocked in buffer B with 1 μg BSA and 1 μg tRNA per μL magnetic amylose resin. After 1 h at 4 °C, the resin was washed 3 times in buffer B and then incubated with lysates from infected cells. NP-40 lysates from wild type PV1 or PV1-3′MS2 infected cells were pre-blocked with equal volume amylose resin for 1 h at 4 °C prior to incubation with MBP-MS2 amylose (1 μL resin per 10 μg lysate). Samples were incubated for 4 h at 4 °C with rotation. The resin was washed 3 times with buffer B and complexes were eluted with 30 mM maltose in buffer B for 30 min at 4 °C. Purified samples were either heated for 1 h at 70 °C for mass spectrometry analysis or LSB was added to samples which were then resolved by sodium dodecyl sulfate-polyacrylamide gel electrophoresis (SDS-PAGE). Gels were analyzed by SYPRO Ruby staining (Lonza, Basel, Switzerland) or proteins were transferred to a PVDF membrane and analyzed by Western blot. For Western blot analysis, antibodies directed against PABP (Santa Cruz Biotechnology, Inc., Dallas, TX, USA), hnRNP C1 + C2 (Abcam, Cambridge, MA, USA), or AUF1 (Millipore, Temecula, CA, USA) were used. For mass spectrometry analysis, eluted samples were digested with trypsin and subjected to nano-liquid chromatography-tandem mass spectrometry (nanoLC-MS/MS). For purification of eluted RNA, 10% of final elution volume was subjected to phenol/chloroform extraction and ethanol precipitation.

### 2.8. S1 and D8 Aptamer Affinity Purification

RNA affinity purification utilizing S1 and D8 aptamers was performed as described in [33,34]. *In vitro* transcribed RNA (11 μg) was renatured in TE buffer (10 mM Tris-HCl pH 7.5, 1 mM EDTA) by heating at 56 °C for 5 min, 37 °C for 10 min, and incubating at room temperature for 15 min. Streptavidin-agarose (SigmaAldrich,) was prepared by washing 10 times in lysis buffer (50 mM HEPES pH 7.4, 10 mM MgCl_2_, 100 mM NaCl, 1 mM DTT, 0.1% Triton X-100, 10% glycerol), Complete protease inhibitors (Roche) and resuspended to 50% slurry in lysis buffer. Sephadex matrix was prepared by swelling 0.5 g Sephadex G-200 (Sigma-Aldrich) in 40 mL lysis buffer overnight at room temperature. The Sephadex was then washed 3 times with lysis buffer and resuspended to 50% slurry. RNA (1 μg) was subjected to electrophoresis on a 1% agarose gel in Tris/Borate/EDTA (TBE) buffer to confirm an intact, homogenous RNA population. The remaining 10 μg of RNA was combined with 100 μL prepared streptavidin beads or Sephadex G-200 and 500 μL of lysis buffer. Samples were allowed to rotate at 4 °C for 4 h then subjected to centrifugation at ~25 rcf for 1 min to separate matrix, and supernatant was removed. Matrices were washed 5 times with 500 μL of lysis buffer, with rotation at 4 °C for 10 min. The matrix slurry was loaded directly onto a 1% TBE agarose gel containing ethidium bromide and subjected to electrophoresis to visualize RNA associated with matrix. As a comparison, the supernatant from the initial matrix separation (*i.e.*, the flow-through) was also subjected to electrophoresis on the agarose gel. Elution of aptamer-tagged RNA was also carried-out by transferring matrix material slurry, following wash steps, to Ultrafree-MC HV centrifugal filter units (0.45 μm pore size (Millipore)) with 10 mM biotin (Sigma-Aldrich) or 50 mg/mL dextran (average molecular weight 9000–11,000 Da (Sigma-Aldrich)) in lysis buffer. Filter units were subjected to rotation at 4 °C for 1.5 h then subjected to centrifugation at ~7500 rcf for 2.5 min.

Isolation of recombinant viral RNA from infected cells was carried-out as described above, but the streptavidin matrix was first blocked with 10 μg avidin from egg white (Sigma-Aldrich). To generate lysates from infected cells, two 150 mm plates of HeLa cell monolayers were infected with wild type PV1, D8+702PV1, or S1+702PV1 at an MOI of 20 following two washes with PBS. After 30 min adsorption, DMEM-8% NCS was added to cells which were then placed in 37 °C incubator for 4 h. Formaldehyde cross-linking was incorporated where indicated as described in [35]: infected cells were washed twice with PBS then cross-linked with 0.2%–1% formaldehyde in PBS for 10 min at room temperature with shaking. Formaldehyde was quenched with 0.25 M glycine for 5 min at room temperature. Infected cells or infected and cross-linked cells were collected by scraping and pelleted at ~1500 rcf for 5 min, followed by washing twice with ice cold PBS. Pellets were resuspended in lysis buffer containing 20 U/μL RNasin (Promega). Cells were lysed by three rounds of sonication for 10 s followed by incubation on ice for 2 min. Lysates were pre-cleared by centrifugation and supernatant was incorporated into the isolation procedure.

## 3. Results

### 3.1. A Recombinant Poliovirus Containing Tandem MS2 Hairpins in Place of the 3′NCR

#### 3.1.1. Recombinant Poliovirus Containing Tandem Bacteriophage MS2 Hairpins in Place of the 3′NCR is Viable and Infectious

**Figure 1:**
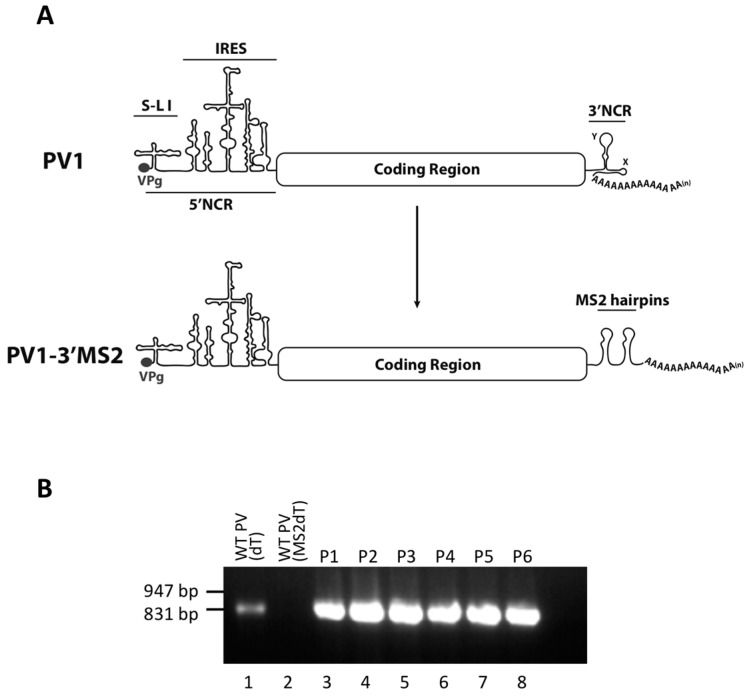
The generation of a recombinant poliovirus containing MS2 hairpins in place of the 3′NCR. (**A**) Schematic of the wild type poliovirus (PV1) genome showing VPg, the 5’ noncoding region (5’NCR), stem-loop (S-L I), and the internal ribosome entry site (IRES). Below the PV1 genome, the genome of the recombinant poliovirus containing tandem MS2 RNA hairpins in place of the 3′NCR (PV1-3′MS2) is shown. (**B**) The tandem MS2 sequence is stable in place of the 3′NCR for up to six passages in cell culture. RT-PCR with primers specific to MS2-poly(A) sequence (MS2dT) was performed to amplify the last 850 nucleotides at the 3′ terminus of viral RNA isolated from HeLa cells infected with PV1-3′MS2 at each of six viral passages (P1–P6) and separated by agarose gel electrophoresis (lanes 3–8). As a positive control, RNA from wild type PV1-infected cells was amplified with poly(dT) primer (dT) to nonspecifically amplify RNA from the 3′ poly(A) tract (lane 1). RNA isolated from wild type PV1-infected was amplified with MS2dT primers and served as a negative control (lane 2). DNA size markers are indicated to the left of the image.

The 3′NCR of the poliovirus genome is not required for viral replication, as a mutant poliovirus lacking this entire region is infectious and stable [29,36]. Thus, our initial studies focused on the generation of recombinant poliovirus containing RNA sequence corresponding to tandem bacteriophage MS2 hairpins, which is of similar length to the 3′NCR itself, in place of the 3′NCR of the genome. The MS2 affinity purification assay is a well-established method for the purification of RNA-protein complexes via the high affinity (K_d_ ~ 1–3 × 10^−9^ M) interaction between the MS2 coat protein and a stem-loop structure within the phage genome, known as the operator or MS2 hairpin, and has been utilized for the purification of RNP complexes associated with long RNAs from cellular extracts [37,38,39]. To generate a recombinant poliovirus containing these RNA affinity sequences within the genome (PV1-3′MS2, Figure 1A), RNA was transcribed *in vitro* and transfected into HeLa cell monolayers with a liquid overlay. Overlays were harvested upon the observation of cytopathic effects and used to infect HeLa cells for plaque purification and screening. Recombinant virus containing the MS2 RNA hairpins was isolated and virus stocks were generated by amplification in cell culture. To confirm the presence of the tandem MS2 tags in the viral genome, RT-PCR with one of two primers directed against the MS2 hairpin sequence was used to amplify the 3’ terminal 850 nucleotides of the viral RNA following each passage in cell culture. The exogenous sequence insertion within PV1-3′MS2 was stable for at least six passages (Figure 1B).

Viral RNA isolated from the third passage of PV1-3′MS2 was subjected to RT-PCR followed by sequencing of the entire genome to determine if mutations had accumulated to compensate for the replacement of the 3′NCR. The only non-synonymous mutation that arose in the genome of PV1-3′MS2 was a phenylalanine-to-serine mutation at amino acid 120 of the capsid protein VP3. This mutation is located at the terminus of one beta strand on the internal side of an 8-stranded anti-parallel beta barrel that forms the poliovirus capsid [40]. It is not clear whether this mutation has any effect on the structure of VP3, since it is located on the surface-exposed beta barrel adjacent to an invariable loop region. An additional tyrosine-to-histidine mutation emerged within 3D^pol^ at amino acid position 15. However, this mutation was also observed following sequencing of the original cDNA used to transcribe RNA for transfection and isolation of virus, and therefore is not a relevant compensatory mutation within the PV1-3’MS2 genome.

#### 3.1.2. PV1-3’MS2 has Delayed Growth Kinetics Compared to Wild Type Poliovirus

**Figure 2:**
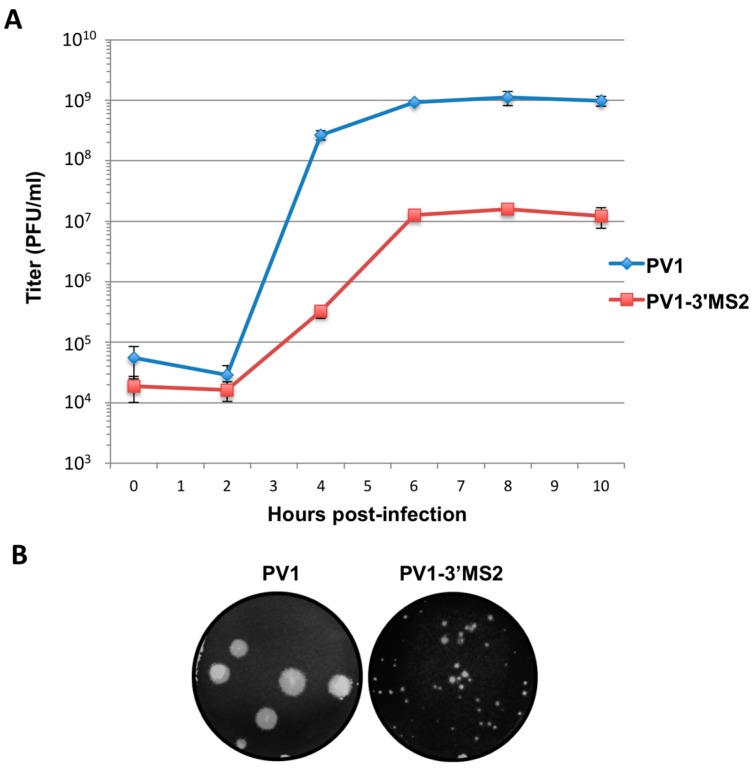
PV1-3′MS2 recombinant virus growth kinetics and plaque morphology. (**A**) A single-cycle growth analysis was carried out in HeLa cell monolayers infected with wild type poliovirus (PV1) or PV1-3′MS2 at an MOI of 20. Cells and supernatant were harvested at 2 h intervals beginning at 0 h post-infection until 10 h post-infection. Virus yield, expressed in plaque forming units per milliliter (PFU/mL) was determined by plaque assay, in triplicate, and plotted. Error bars represent one standard deviation above and below the mean. (**B**) To observe plaque morphology, HeLa cell monolayers seeded on 60 mm dishes were infected with wild type poliovirus (PV1) or PV1-3′MS2 and overlaid with semi-solid medium containing agarose. After 4 days of incubation at 37 °C, cells were fixed with 10% trichloroacetic acid and stained with crystal violet to visualize plaques. Plaque sizes were measured, and PV1-3′MS2 was determined to produce plaques approximately 70% smaller than wild type poliovirus.

To characterize the growth properties of the recombinant PV1-3′MS2 virus, a single-cycle growth analysis was performed and compared to the same analysis of wild type poliovirus (Figure 2A). Third passage stocks of PV1-3′MS2 or wild type poliovirus were used to infect HeLa cell monolayers, and cells and cell culture fluids were collected every 2 h to measure virus production over the course of infection. The PV1-3′MS2 recombinant virus showed a 2 log_10_ reduction in viral titer and a 2 h delay in peak virus production, which occurs at around 4 h post-infection for wild type poliovirus. The growth deficiency in the genetically-tagged virus was also evidenced by the small plaque phenotype of PV1-3′MS2 when compared to wild type poliovirus (Figure 2B). Because a mutant poliovirus lacking the 3′NCR sequence within its genome (Δ3′NCR PV1) demonstrates a similar growth defect compared to wild type poliovirus, the lack of the 3′NCR sequence is likely responsible for the limited virus production from PV1-3′MS2, rather than the presence of the tandem MS2 RNA sequence itself [36]. Furthermore, negative-sense RNA levels produced during infection with Δ3′NCR PV1 are comparable to levels observed during wild type poliovirus infection, but Δ3′NCR PV1 synthesizes significantly lower levels of positive-sense RNA compared to wild type [36]. By extension, we predict that the growth defect observed during PV1-3′MS2 infection is a result of limitations to positive-strand RNA production as a result of the negative-strand template RNA lacking the 5′ terminal region, the complement to the positive-strand 3′NCR.

**Figure 3:**
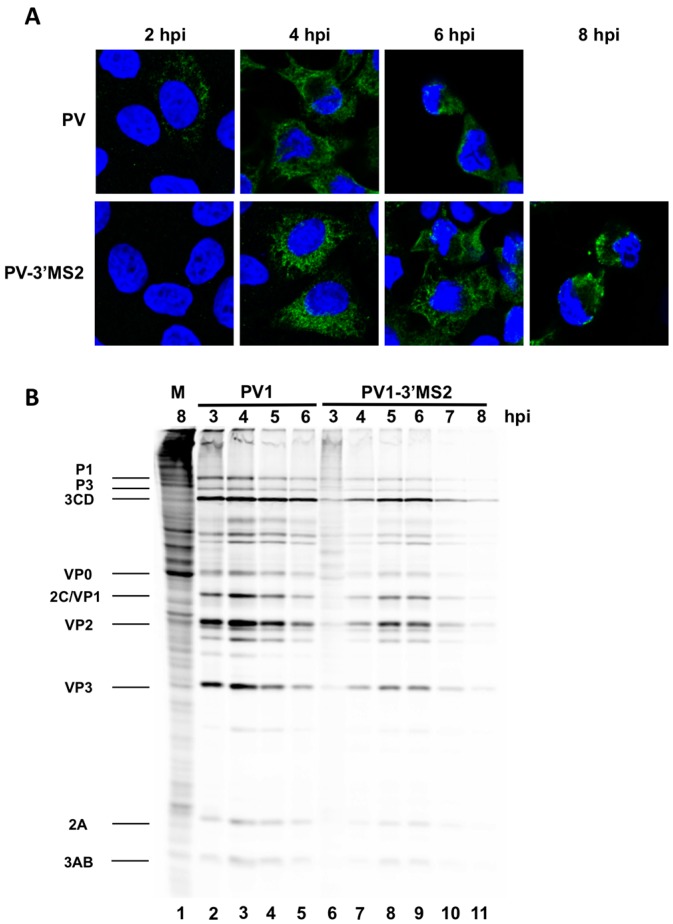
PV1-3’MS2 displays a delay in viral protein production and cytopathic effects. (**A**) HeLa cells seeded on coverslips were infected with wild type poliovirus (PV1) or PV1-3′MS2 at an MOI of 20 and fixed with formaldehyde at the indicated times post-infection. Cells were immunostained with anti-3A antibody (green), stained with DAPI (blue) to indicate the nucleus, and visualized by confocal microscopy. (**B**) HeLa cell monolayers were mock-infected (M, lane 1), infected with wild type poliovirus (PV1, lanes 2–5), or infected with PV1-3′MS2 (lanes 6–11) and incubated in methionine-free media. One hour prior to collection, ^35^S-methionine was added to label translation products. At the indicated hour post-infection (hpi), cells were harvested and boiled in LSB. Twenty-five percent of total reactions were subjected to SDS-PAGE and autoradiography. Viral proteins are labeled to the left of the image.

If positive-sense RNA synthesis was indeed the cause of the delay in virus production during PV1-3′MS2 infection observed in Figure 2A, then the production of viral proteins from these RNAs, which are also templates for translation, would also be delayed in cells infected with PV1-3′MS2. Viral protein production was examined via immunofluorescence detection of the viral protein 3A in cells infected with PV1-3′MS2 or wild type poliovirus (Figure 3A). Viral protein 3A and its precursor 3AB are nonstructural proteins involved, in part, in the formation of membranous structures that are the sites of viral RNA replication. In wild type poliovirus-infected cells, 3A was detected by 2 h post-infection, with increased production of 3A observed 4 h post-infection. Deterioration of the nucleus, detected at 6 h post-infection with wild type poliovirus, is representative of cytopathic effects. In contrast, the presence of viral protein 3A was not detected in cells at 2 h post-infection with PV1-3′MS2, and 3A is not observed in infected cells until 4 h post-infection, reflecting the 2 h delay in virus growth observed by single-cycle growth analysis. Furthermore, cytopathic effects are not observed until 8 h post-infection with PV1-3′MS2. Accumulation of viral translation products was also examined over the course of infection by ^35^S-methionine labeling of viral proteins during the infectious cycle (Figure 3B). Because poliovirus infection results in the inhibition of cap-dependent translation through cleavage of canonical translation initiation factors, cellular protein production is inhibited in infected cells, resulting in the incorporation of radiolabeled methionine only in viral proteins (compare background proteins in lane 1 to lane 2). Viral proteins are readily apparent by 3 h post-infection with wild type poliovirus, but very few viral proteins are detected by 3 h post-infection with PV1-3′MS2. Additionally, complete shut down of cellular translation is delayed until 4 h post-infection with recombinant virus (compare Figure 3B lane 1 to lanes 6 and 7), confirming the delay in viral processes observed by single-cycle growth analysis and immunofluorescence microscopy.

#### 3.1.3. MS2 RNA Affinity Purification of Viral RNA

**Figure 4:**
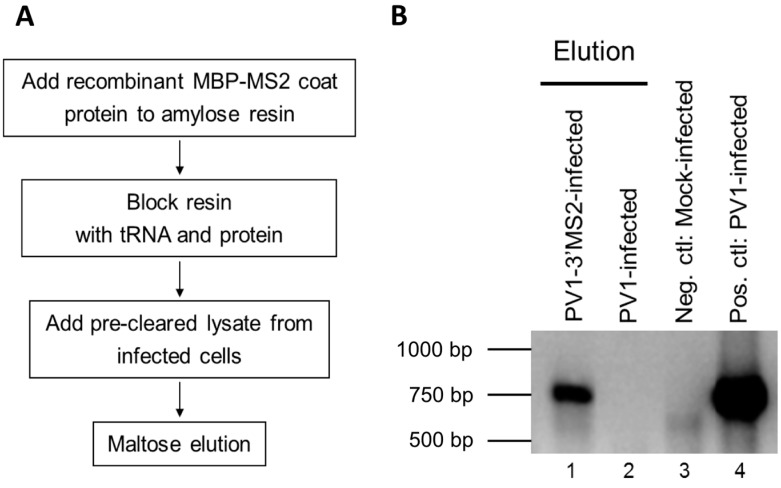
Purification of PV1-3VMS2 RNA by MBP-MS2 coat protein affinity. (**A**) Diagram of MBP-MS2 RNA affinity purification procedure. First, recombinant MBP-MS2 coat protein was added to amylose beads to create the resin to bind viral RNA containing MS2 hairpins. This resin was then blocked to prevent nonspecific interactions. NP-40 lysate from infected cells was pre-cleared with amylose beads and added to the MBP-MS2 amylose beads. Following three washes, MBP-MS2 and bound complexes were eluted from the amylose beads with maltose buffer. (**B**) Subsequent to MBP-MS2 purification from lysates of wild type poliovirus- (PV1, lane 2) or PV1-3′MS2- (lane 1) infected cells, eluted samples were subjected to phenol-chloroform extraction, and RNA was ethanol precipitated. RT-PCR was performed using primers specific for poliovirus positive-sense RNA, and products were resolved by agarose gel electrophoresis. RT-PCR products amplified from RNA isolated from mock-infected cells served as a negative control for the poliovirus specific primers (lane 3). RNA extracted from wild type poliovirus-infected cells was used as a positive control for RT-PCR with poliovirus primers (lane 4). DNA size markers are indicated to the left of the image.

Following the confirmation of the genetic stability of the PV1-3′MS2 genome and determination of the growth characteristics of this recombinant virus, maltose binding protein (MBP)-MS2 affinity purification was performed to isolate MS2-tagged viral RNA. Lysates of PV1-3′MS2 infected cells were generated by harvesting cells 6 h post-infection, 2 h later than the peak of RNA synthesis in wild type poliovirus, to compensate for the 2 h growth delay observed with the PV1-3′MS2 virus. In an effort to increase the detection of transient and dynamic protein-RNA interactions that occur during infection, infected cells were subjected to 0.4% formaldehyde cross-linking prior to the generation of NP-40 lysates. To generate MS2 affinity resin for purification of MS2-tagged viral RNA, recombinant MS2 coat protein fused to maltose binding protein (MBP-MS2) was incubated with amylose resin and subsequently blocked with tRNA and bovine serum albumin to limit nonspecific interactions (Figure 4A). Pre-cleared NP-40 lysates from infected cells were incubated with blocked MBP-MS2 bound to amylose resin and incubated for 4 h. MBP-MS2 and bound RNA with associated proteins were eluted from the amylose resin with maltose. Lysates from wild type poliovirus-infected cells were generated and subjected to the same purification scheme as a negative control. Reverse transcription followed by PCR amplification with primers specific for the coding region of poliovirus was performed to verify the purification of PV1-3′MS2 RNA (Figure 4B). Poliovirus cDNA amplified by RT-PCR from eluted fractions was only present in lysates from PV1-3′MS2-infected cells, suggesting that wild type RNA does not interact with the MS2 coat protein (Figure 4B, compare lane 1 and lane 2). These results demonstrate the functional use of MS2-tagged RNA affinity purification for isolating the recombinant poliovirus PV1-3′MS2 genome from infected cells.

#### 3.1.4. Composition of RNP Complexes Isolated from PV1-3′MS2-Infected Cells via MS2 RNA Affinity Purification

**Figure 5:**
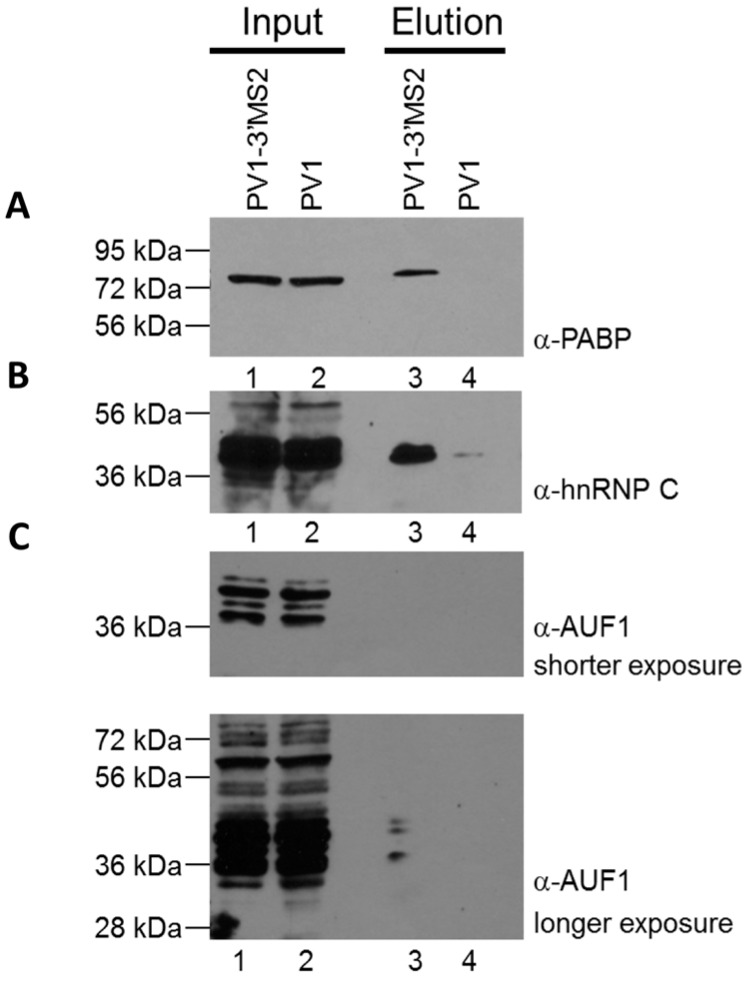
Cellular proteins with known roles in poliovirus replication co-purify with PV1-3′MS2 RNA. Eluted samples from wild type poliovirus and PV1-3′MS2 RNA purifications were analyzed by SDS-PAGE and Western blot with antibodies directed against PABP1 (**A**), hnRNP C1/C2 (**B**), or AUF1 (**C**), short exposure on top panel, long exposure on bottom panel). Lanes 1 and 2 represent 10% of experimental input. Lane 3 represents purifications from lysates of PV1-3′MS2-infected cells collected at 6 h post-infection and lane 4 is purifications from lysates of wild type poliovirus-infected cells collected 4 h post-infection. Molecular weight markers are indicated on the left of the images.

To confirm that MBP-MS2 affinity purification of PV1-3′MS2 RNA allowed for co-isolation of known components of poliovirus RNPs, purified fractions were subjected to SDS-PAGE and Western blot analysis. Three proteins known to bind poliovirus RNA were enriched in purified samples from PV1-3′MS2-infected cells compared to purifications from wild type poliovirus-infected cells (Figure 5A–C; compare lane 3 to lane 4). The cellular protein PABP1 was specifically purified in association with PV1-3′MS2 (Figure 5A), and has been proposed to play a role in viral replication via its interaction with the poly(A) tract of the poliovirus genome [22]. Another protein with a role in poliovirus RNA synthesis that was enriched by MS2 purification, hnRNP C1/C2, has been shown to interact with RNA sequences corresponding to both the 5′ and 3′ terminal regions of negative-sense poliovirus RNA (Figure 5B) [25,27]. Although hnRNP C1/C2 binding to positive-sense poliovirus RNA molecules has not been demonstrated, it may be purified from infected cells through isolation of partially double-stranded replicative form or multi-stranded replicative intermediate complexes containing both polarities of poliovirus RNA, which form during viral RNA replication. Furthermore, a recently identified negative regulator of poliovirus replication, AU-rich element RNA-binding protein 1 (AUF1, also known as heterogeneous nuclear ribonucleoprotein D0), was also co-purified with PV1-3′MS2 RNA, albeit at low levels, presumably through its interaction with the 5′NCR of poliovirus RNA [41]. These results demonstrate that isolation of PV1-3′MS2 RNA from infected cells allows for the enrichment of proteins known to interact with poliovirus RNA molecules and which carry out distinct functions within the viral RNA replication cycle.

**Figure 6:**
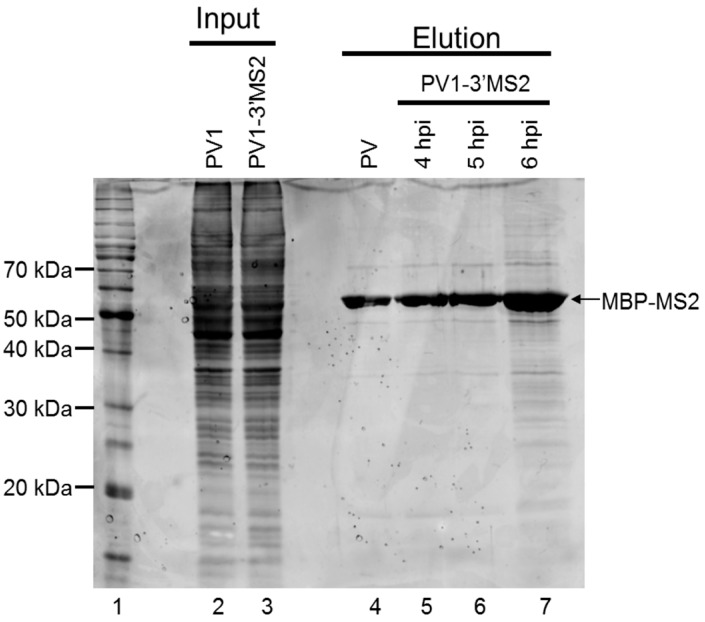
Analysis of proteins isolated by MS2 affinity purification. Samples purified as described in Figure 4A were analyzed by SDS-PAGE and SYPRO Ruby staining. Lane 1 shows molecular weight markers, which are labeled on the left of the image. Lanes 2 and 3 represent 10% of experimental input from lysates generated from cells infected with wild type poliovirus collected 4 h post-infection (hpi) and from PV1-3′MS2-infected cells collected at 6 h post-infection, respectively. Lane 4 corresponds to purified sample from lysates of wild type poliovirus-infected cells collected at 4 h post-infection, the negative control for assaying for the specificity of the isolation procedure. Lanes 5–7 represent samples eluted during MS2 affinity purification of PV1-3′MS2-infected cells collected at 4, 5, and 6 h post-infection, respectively. The electrophoretic mobility of MBP-MS2 is indicated on the right side of the image.

To examine the protein complexity present within purified samples following MS2 affinity purification, eluted fractions were separated by SDS-PAGE and stained with SYPRO Ruby (Figure 6). Because the replication cycle of PV1-3′MS2 is delayed by 2 h (Figure 3), lysates from infected cells were generated at 4, 5, and 6 h post-infection to make up for this delay as well as to identify changes in RNP composition that may reflect different stages of the viral replication cycle. We observed some nonspecific isolation of proteins associated with the MS2 affinity purification of wild type RNA and associated proteins from infected cells (Figure 6, lane 4). However, an increased amount of co-purified proteins was detected in samples from PV1-3′MS2-infected cells at 5 h post-infection, with even higher levels of co-purified proteins observed by 6 h post-infection (Figure 6, lanes 6 and 7). Whether the apparent increase in the amount and diversity of proteins enriched upon PV1-3′MS2 RNA purification represents an authentic increase in the diversity of proteins that make up poliovirus RNP complexes at later times during infection, or mainly a consequence of increased abundance of viral RNA and consequent increases in co-isolated proteins, remains to be determined. In an attempt to characterize the complete composition of the RNP complexes isolated via the MS2 RNA hairpins present in the PV1-3′MS2 genome, purified fractions were digested with trypsin and peptides were identified by mass spectrometry. Results from purification of lysates from PV1-3′MS2-infected cells were compared to results from purification of lysates from wild type poliovirus-infected cells to verify specific interactions. Analysis of those proteins purified from wild type poliovirus-infected cells revealed a high background of nonspecific interactions using our current purification procedure. Mass spectrometry analysis demonstrated that the total number of proteins identified as well as the relative composition of proteins categorized by molecular function were nearly identical regardless of the presence of the tandem MS2 hairpin RNA tag during purification (data not shown). Proteins nonspecifically purified from lysates of both recombinant and wild type poliovirus-infected cells include components of the 40S and 60S ribosomal subunits, serine/arginine-rich splicing factors, and peptides corresponding to viral proteins. Taken together, these results suggest that our MS2 RNA affinity purification needs to be further refined to allow for the identification of cellular components that function in poliovirus RNA replication, especially relative to the sensitivity of mass spectrometry analysis.

### 3.2. Recombinant Polioviruses Containing RNA Aptamers within the 5′NCR

#### 3.2.1. Recombinant Poliovirus RNAs Containing Aptamer Sequences Produce *in vitro* Translation Products Equivalent to Those Produced by Wild Type Poliovirus RNA

As a result of the lack of specificity we observed during purifications of poliovirus RNP complexes associated with PV1-3′MS2 RNA via MS2 affinity, we pursued alternate RNA affinity tags for the isolation of recombinant viral genomes and accompanying proteins. Aptamers developed to specifically bind streptavidin or Sephadex, a cross-linked dextran gel used in size-exclusion chromatography, were selected as affinity sequences for subsequent purifications. These RNA sequences are short: the consensus S1, streptavidin-binding aptamer is 44 nucleotides in length and the D8, Sephadex-binding aptamer is 33 nucleotides long [42,43]. Because large sequence insertions presumably incur a fitness cost on recombinant viral genomes, and because we were interested in generating viruses with as close to wild type replication kinetics as possible, the length of the sequence was a major determinant in our selection of affinity tag. These aptamer tags were also appealing because of their potential for RNP complex isolation and subsequent characterization, as has been previously demonstrated for cellular mRNAs and rRNAs [44,45,46,47,48,49,50,51].

**Figure 7:**
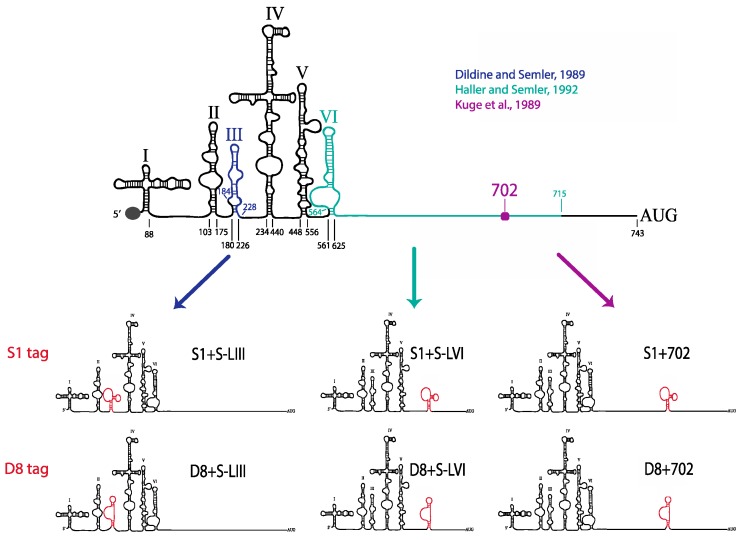
Schematic of poliovirus genomic RNAs containing S1 and D8 aptamer tags within the highly structured 5′NCR, for positive-strand isolation. Insertion of either the S1 streptavidin-binding or D8 Sephadex-binding aptamer sequence within three regions of the poliovirus 5′NCR resulted in the production of 12 poliovirus cDNA constructs. Tag insertions in place of S-L III (blue), in place of S-L VI (green), or at nucleotide position 702 (purple) are depicted on the left, middle, and right, respectively. Constructs for negative-strand isolation (aptamers inserted in a reverse orientation) are not shown.

There are four known regions within the 5′NCR of the poliovirus genome that have previously been shown to tolerate major sequence alterations: S-L III, S-L VI, nucleotide position 600 through 726 (including a portion of S-L VI), and just downstream of nucleotide position 702 [30,52,53,54]. Deletions of these S-L regions or insertions of up to 72 nucleotides at position 702 produces mutant viruses that have essentially wild type growth kinetics and yields, with only slight delays in RNA synthesis. Based on the results of these previous studies, we separately introduced the minimal D8 and S1 motifs into three different locations within the poliovirus 5′NCR: in place of S-L III, in place of S-L VI, or at nucleotide position 702 (Figure 7). These aptamer tags were engineered into these sites in a forward or reverse orientation within a poliovirus cDNA construct, subsequently allowing for the D8 or S1 RNA sequence motif to be present in the viral positive- or negative-strand RNA, respectively, potentially allowing for strand-specific RNP complex isolation. This resulted in the production of 12 plasmid constructs: three constructs harboring the D8 tag at the three separate sites for positive-strand isolation (D8+S-LIII, D8+S-LVI, D8+702), three constructs harboring the D8 tag for negative-strand isolation (D8−S-LIII, D8−S-LVI, D8−702), and the corresponding six constructs containing the S1 nucleotide sequence (S1+S-LIII, S1+S-LVI, S1+702, S1−S-LIII, S1−S-LVI, S1−702).

**Figure 8:**
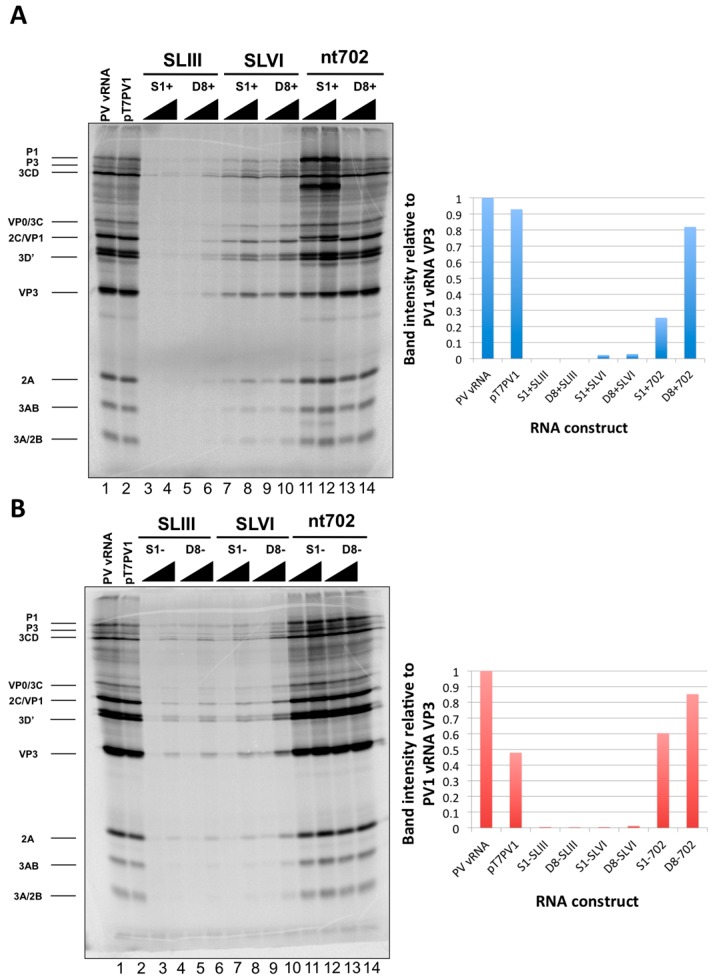
*In vitro* translation assays of poliovirus RNA constructs containing aptamer tags within the 5′NCR. *In vitro* transcribed recombinant poliovirus RNA molecules containing RNA affinity tags for positive-strand isolation (**A**) or negative-strand isolation (**B**) were subjected to an *in vitro* translation assay utilizing HeLa cell cytoplasmic extracts and ^35^S-methionine to label viral proteins. Translation products from wild type poliovirus RNA isolated from poliovirus virions (PV vRNA) and an *in vitro* transcribed poliovirus cDNA construct containing a T7 promoter (pT7PV1) are shown in lanes 1 and 2, respectively. Constructs containing either the S1 or D8 aptamer tags in place of S-L III, S-L VI, or at position 702 of the genome are shown in lanes 3–14, with either 250 or 500 ng of RNA (increasing RNA amounts are indicated with triangles). Viral translation products are indicated on the left of the images. Quantification of the intensity of the VP3 band observed in assays containing 250 ng of each RNA construct relative to PV vRNA is shown adjacent to each autoradiograph.

Recombinant poliovirus cDNAs were linearized and transcribed *in vitro* to generate RNA corresponding to full-length poliovirus RNA with incorporated RNA affinity tag sequences. Tagged RNAs were subjected to an *in vitro* translation assay, making use of HeLa cell cytoplasmic extracts in the presence of ^35^S-methionine to produce radiolabeled viral proteins. Only those constructs containing affinity tag sequence at nucleotide position 702 produced levels of viral proteins similar to wild type poliovirus RNA (Figure 8). In agreement with previous studies that suggest the introduction of an AUG triplet into the highly variable region between S-L VI and the start codon of the poliovirus genome is deleterious for viral replication, the S1+702 construct, which contains an AUG triplet in-frame with the translation start codon at position 743, showed an altered protein profile compared to wild type poliovirus RNA translation [54]. This in-frame AUG triplet likely leads to alterations in authentic translation initiation, producing amino-terminal extensions on viral proteins P1, 1ABC, and VP0. Interestingly, however, the presence of an AUG triplet within the sequence of the S1 tag for negative-strand isolation, which is out-of-frame with the initiator codon, does not cause alterations to viral protein production compared to wild type RNA sequence.

#### 3.2.2. Recombinant Polioviruses Containing Aptamer Tags at Genomic Position 702 of the 5′NCR are Viable and Have Growth Kinetics Indistinguishable from Those of Wild Type Poliovirus

Because only those RNA constructs containing aptamer tag sequence insertions at nucleotide position 702 of the 5′NCR produced viral proteins in amounts similar to wild type poliovirus within the *in vitro* translation assay, these constructs were transfected into HeLa cell monolayers for the generation of recombinant virus. As with the generation of PV1-3′MS2, virus stocks were generated following plaque purification and amplification in cell culture. Recombinant viral genomes were subjected to RT-PCR with primers designed to amplify the 5′NCR and sequenced to examine the stability of the tag insertions after the second and third passage in cell culture. D8+702PV1, D8−702PV1, and S1-702PV1 viruses contained intact aptamer sequence after both the second and third passage in cell culture, with no other alterations to the 5′NCR. The S1+ 702PV1 recombinant virus, in contrast, did not retain the S1 streptavidin affinity tag sequence without alterations. The in-frame AUG triplet within the S1 aptamer sequence was selected against during infection, with three distinct alterations to the AUG triplet observed in four separate isolates. Importantly, only the adenosine residue of the AUG triplet within the S1 aptamer sequence is conserved within the consensus sequence of the multiple streptavidin-binding aptamer sequences first identified [42]. Thus, a viral isolate containing a single nucleotide change, a guanosine-to-uridine transversion at the third position of the triplet (S1+702AUUPV1), was selected as the recombinant virus possessing a streptavidin-binding genome to use in subsequent assays. This single nucleotide alteration within the S1 tag was stable within the poliovirus genome through three passages in cell culture, demonstrated by sequencing progressive passages of virus isolates. *In vitro* translation assays incorporating an S1+702 *in vitro* transcribed RNA containing the transversion observed within the S1+702AUUPV1 genome showed a wild type protein profile for this altered construct (data not shown), supporting the idea that the insertion of an in-frame AUG is unfavorable for viral protein production. We also generated recombinant virus from the RNA construct containing the D8 tag for negative-strand isolation in place of stem-loop VI. However, after a single passage in cell culture, sequencing cDNA generated from viral genomes revealed over half of the nucleotides within the tag sequence had been deleted, as well as 20–25 nucleotides of the genome downstream of the insertion site.

**Figure 9:**
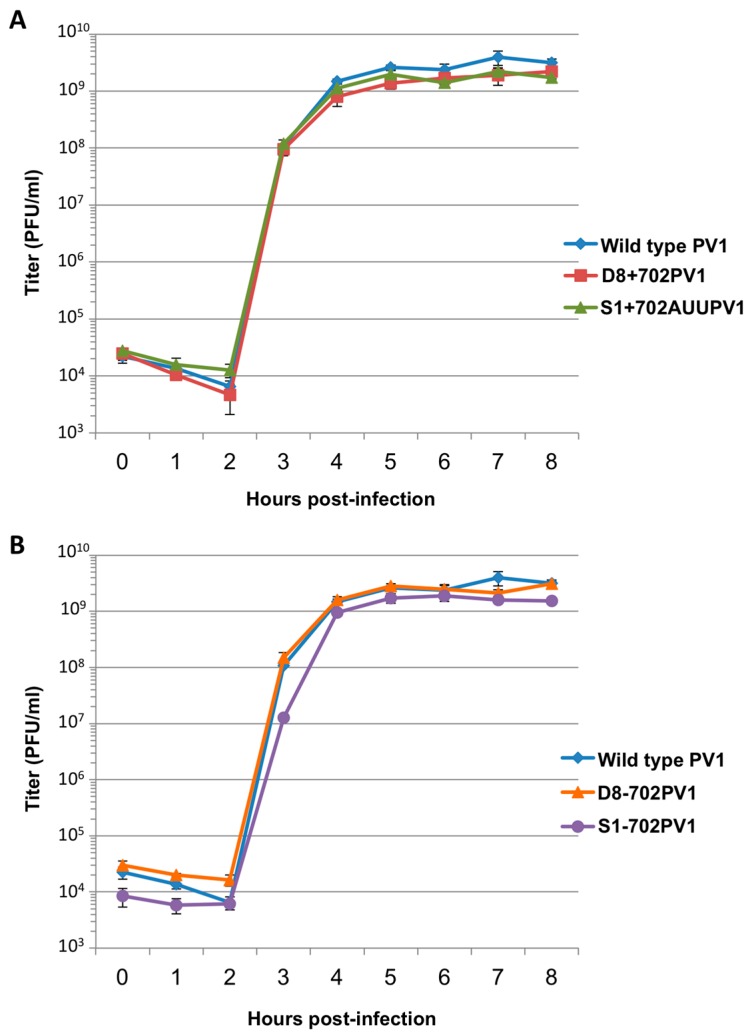
Single-cycle growth kinetics of recombinant polioviruses containing aptamer tag sequence at nucleotide position 702 of the 5′NCR. (**A**) A single-cycle growth analysis was carried out in HeLa cell monolayers infected with wild type poliovirus (PV1) or recombinant viruses containing aptamer tag for positive-strand isolation (D8+702PV1 or S1+702AUUPV1) at an MOI of 20. Cells and supernatant were harvested at 1 h intervals beginning at 0 h post-infection until 8 h post-infection, in triplicate. Virus yield was determined by plaque assay and plotted. (**B**) The same analysis as in (**A**) but comparing recombinant viruses containing aptamer tag for negative-strand isolation (D8-702PV1 or S1-702PV1) to wild type poliovirus. Titer is given in plaque forming units (PFU) per milliliter. Error bars represent one standard deviation above and below the mean.

To characterize the growth properties of these recombinant viruses, single-cycle growth analyses were performed. Third passage stocks of virus were used to infect HeLa cell monolayers, then liquid overlay and cells were collected at intervals and subjected to plaque assays to determine viral titers. The single-cycle growth curves demonstrate that all recombinant viruses have essentially wild type growth kinetics, with maximum virus production reached 4 h following infection (Figure 9). No significant differences in plaque morphology between wild type poliovirus and recombinant viruses containing RNA affinity tag sequences were observed (data not shown). Furthermore, complete sequencing of third passage stocks of these recombinant viral genomes demonstrated that only a handful of changes to the third position of codons had occurred, resulting in synonymous mutations to amino acid sequences. A threonine-to-isoleucine mutation at amino acid position 93 of 3D^pol^ was also identified, but this alteration was also present in wild type poliovirus generated from transfections of the pT7PV1 cDNA backbone, and therefore not a mutation compensatory for the presence of aptamer tag within the 5′NCR.

#### 3.2.3. Poliovirus cDNA Constructs Transcribed *in Vitro* Can Be Enriched via RNA Affinity Tags but Recombinant Viral RNA Isolation from Infected Cells is Inefficient

**Figure 10:**
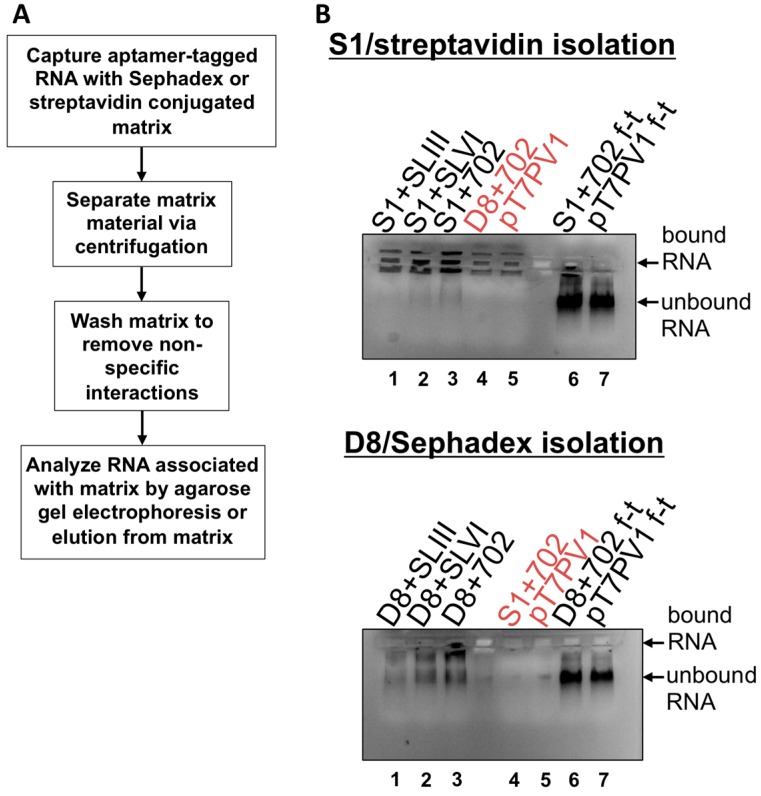
*In vitro* transcribed affinity-tagged RNA isolation. (**A**) Diagram of the RNA isolation procedure based on the use of S1 and D8 aptamer tags. Aptamer-tagged RNAs were captured by the respective matrix via incubation for 4 h at 4 °C, under rotation. The matrix material was then separated by centrifugation and washed 3–5 times to remove non-specific associations to the matrix. Finally, the matrix material was analyzed by agarose gel electrophoresis or incubated with dextran or biotin to elute aptamer-tagged poliovirus RNA. (**B**) Matrix material and associated RNA was loaded on a 1% agarose gel containing ethidium bromide and subjected to electrophoresis. Relative levels of RNA association are depicted as increased staining intensity within the wells of the agarose gel. Red labels of lanes 4 and 5 indicate negative controls: either wild type RNA (pT7PV1) or RNA containing an irrelevant tag for the matrix. Lanes 6 and 7 labeled as “f-t” are flow-through fractions that were loaded with the supernatant removed following the initial centrifugation, prior to the first matrix wash step. Unbound RNA migrates into the gel itself. Levels of unbound RNA present in samples removed following coupling step were similar between aptamer-tagged RNAs and wild type RNA, demonstrating the limited binding efficiency of these aptamers within the context of the 7.5 kb poliovirus RNA.

After confirming the stability of affinity tag sequences within viral genomes and characterizing the growth kinetics of recombinant polioviruses, affinity purification procedures were tested. Aptamer-tagged RNA isolation was first performed *in vitro*, as described in Walker, *et al.*, 2008 [33] (Figure 10A). *In vitro* transcribed poliovirus RNAs for positive-strand isolation were generated and renatured by heating and slow cooling. Aptamer binding was carried out under rotation at 4 °C for 4 h, followed by separation of matrix material through centrifugation, and then the Sephadex or streptavidin-conjugated bead matrix was washed multiple times. RNA associations with insoluble matrix were determined by direct loading of the matrix onto an agarose gel containing ethidium bromide and performing electrophoresis (Figure 10B). The specificity of each construct containing the relevant aptamer tag insertion parallels the level of viral proteins produced in the *in vitro* translation assay shown in Figure 8. This suggests that not only is the nucleotide position 702 insertion site most favorable for the generation of a viable, genetically-tagged virus, but also for matrix accessibility and, as a result, most efficient isolation. Importantly, non-specific interactions between the RNA containing an irrelevant affinity sequence and no affinity sequence (*i.e.*, wild type RNA) were limited (lanes 4 and 5 in both panels of Figure 10B), with the Sephadex matrix showing less background RNA associations than streptavidin beads. Although there appears to be specific association between aptamer-tagged RNAs and the respective matrix, the binding efficiency of these aptamers is very low, as the flow-through from tagged RNA purifications contains similar amounts of RNA compared to untagged, wild type RNA (see lanes 6 and 7 in Figure 10B). Furthermore, elution of tagged RNAs from either matrix is limited, likely due to the minimal amount of tagged RNA associated with the matrix following binding and wash steps (data not shown).

Subsequently, we attempted isolations of affinity-tagged viral RNA from infected HeLa cells. Third passage stocks of recombinant viruses containing aptamer tags for positive-strand isolation were used due to the greater abundance of positive-sense RNA relative to negative-sense RNA in infected cells. Wild type virus infections were used as controls. At 4 h post-infection, cells were harvested and infected cell pellets were resuspended and lysed. Pre-cleared lysates were purified as described above. Although we were able to observe specific isolation of genomes containing S1+ or D8+ tags compared to wild type poliovirus, the binding and elution efficiency associated with these RNAs was reduced compared to the *in vitro* transcribed RNA constructs, and no proteins co-isolated with viral RNA were detected in elution fractions (data not shown). In addition, during infections with recombinant polioviruses, a formaldehyde cross-linking step was tested [35]; however, formaldehyde cross-linking abolished enrichment of recombinant viral genomes (data not shown).

#### 3.2.4. Recombinant Viral Genomes Containing Modified S1 or D8 Aptamer Tags

**Figure 11:**
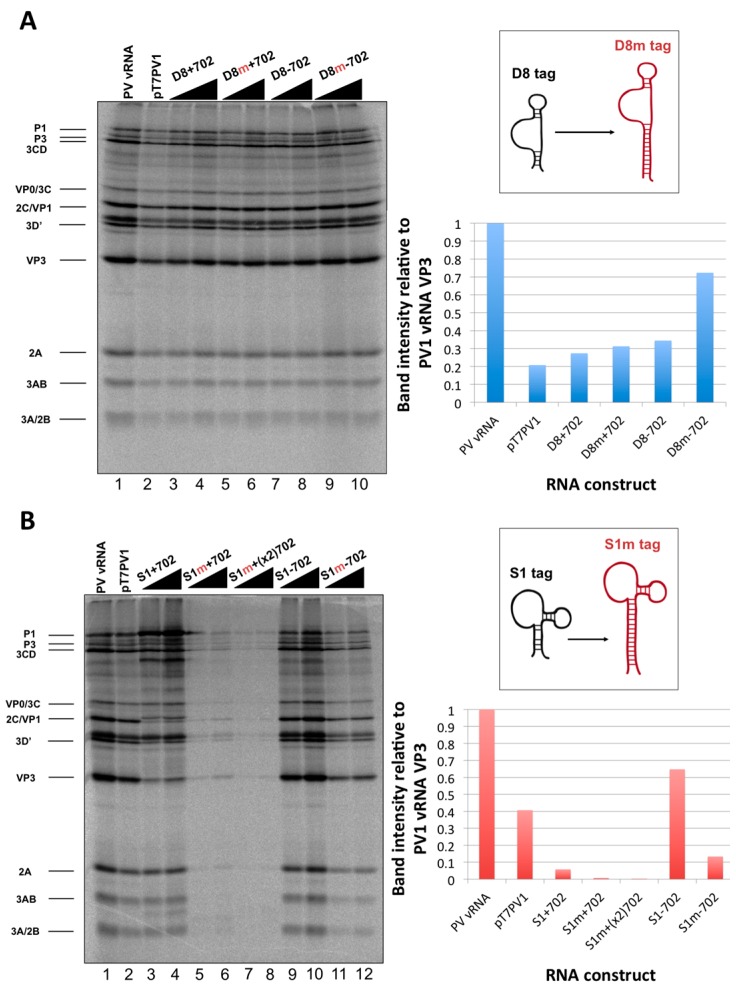
*In vitro* translation of poliovirus RNAs containing modified aptamers for RNA affinity isolation. (**A**) *In vitro* translation of RNA constructs containing original or modified D8 (denoted by a red ”m”) aptamers at nucleotide position 702 of the 5′NCR show very similar levels of protein production compared to wild type RNA (lane 1 and 2). Quantification of the intensity of the band corresponding to VP3, relative to that of poliovirus virion RNA, is shown on the right hand side of the panel. A schematic of the alterations to the tag itself is shown above the quantification. (**B**) *In vitro* translation of RNA constructs containing original or modified S1 aptamers at nucleotide position 702 of the 5′NCR show variable levels of protein production. The RNA construct containing modified S1 tag for positive-strand isolation (lanes 5 and 6) and an RNA construct containing two modified S1 tags in series (lanes 7 and 8) show very little protein production, but the RNA construct containing modified S1 tag for negative-strand isolation (lanes 11 and 12) translates at an intermediate level compared to the original S1 tag-containing constructs. Quantification of the intensity of the band corresponding to VP3 is shown on the right hand side of the panel, and a schematic of the alterations to the tag itself is shown above the quantification. Increasing RNA amounts (250 to 500 ng) are indicated with triangles above the autoradiographs. Bands corresponding to viral protein products are labeled.

In an attempt to increase the limited binding efficiency of S1 and D8 aptamers that we and others have observed [34], we engineered viral genomes with single S1 or D8 tags (in forward and reverse orientations for strand-specific isolation) that contain increased stem length to promote aptamer/matrix interactions for isolation of RNPs. The modified S1 and D8 tags (S1m and D8m) are 60 and 49 nucleotides in length, respectively. RNA constructs containing S1m or D8m at nucleotide position 702 of the genome were generated and characterized as previously described. RNA constructs were first subjected to an *in vitro* translation assay to examine viral protein production from these RNAs (Figure 11). All modified D8 tag-containing constructs translated with similar efficiencies compared to the original D8 containing constructs and wild type poliovirus RNA (Figure 11A). Conversely, constructs containing the modified S1 containing tags, including a construct containing two S1m aptamers in series (S1m+(x2)702), produced limited levels of translation products (Figure 11B). Unlike the minimal S1 aptamer tags, no in-frame AUG sequences were present within these tags, so the limited protein production is likely a result of an inhibitory activity associated with ribosomal recognition or scanning.

**Figure 12:**
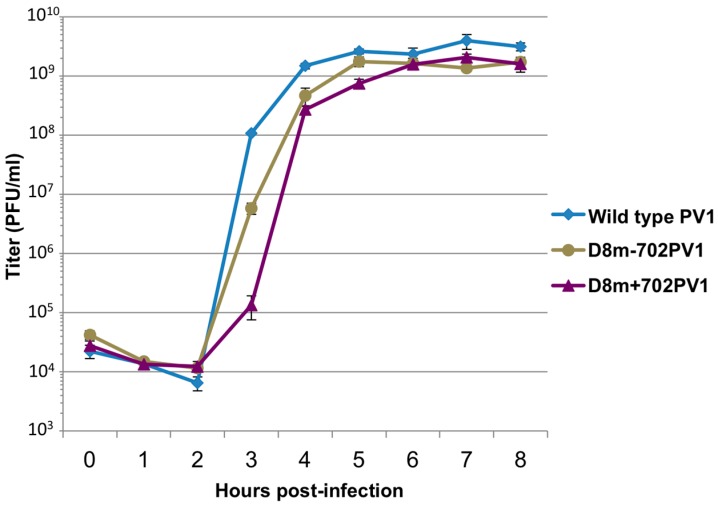
Single-cycle growth analysis of recombinant polioviruses containing modified D8 affinity tags. A single-cycle growth analysis was carried out in HeLa cell monolayers infected with wild type poliovirus (PV1), recombinant virus containing modified aptamer tag for positive-strand isolation (D8+m702PV1), or recombinant virus containing modified aptamer for negative-strand isolation (D8m-702PV1) at an MOI of 20. Cells and supernatant were harvested at 1 h intervals beginning at 0 h post-infection until 8 h post-infection, in triplicate. Virus yield was determined by plaque assay and plotted. Titer is given in plaque forming units (PFU) per milliliter. Error bars represent one standard deviation above and below the mean.

Based on the translation product profiles, we focused on genomes containing modified Sephadex-binding tags for subsequent work. Recombinant polioviruses containing modified D8 aptamer tags in the forward or reverse orientation were generated as previously described. *In vitro* transcribed RNAs were transfected into HeLa cells and resulting recombinant viruses were plaque purified and amplified in cell culture. The stability of the tag sequence was verified by performing RT-PCR followed by sequencing of the 5′NCR of these viruses. As was observed with viruses containing the original D8 tag, the D8m+702PV1 and D8m-702PV1 recombinant polioviruses retained an intact tag within the 5′NCR with no alterations after three passages in cell culture, indicating that these genomic insertions were stable. The growth kinetics of these viruses were then characterized via single-cycle growth analyses (Figure 12). The recombinant polioviruses containing D8 tags of increased length had slight delays in virus production, but very similar titers were observed by the end of the infectious cycle compared to wild type poliovirus. These results suggested that recombinant polioviruses containing modified Sephadex-binding aptamers at nucleotide position 702 of the 5′NCR could be used for the identification of RNP complexes associated with poliovirus RNA replication during the infectious cycle. However, attempts to isolate D8m+702PV1 RNA following infection have not yet been successful.

## 4. Discussion

In an effort to isolate intact enteroviral RNA replication complexes from infected cells at different times following infection and to identify the novel cellular components of these complexes that correspond to the discrete steps of the RNA replication process, we have generated recombinant polioviruses containing genetically encoded RNA affinity tags.

Despite promising results related to the co-isolation of host proteins known to be involved in the replication cycle of poliovirus through MS2 affinity purification of viral RNA, the recombinant PV1-3′MS2 proved to be inadequate for the identification of novel host proteins involved in poliovirus RNA replication. This was in large part due to the sensitivity of mass spectrometry analysis, as significant co-isolation of proteins from negative control purifications (*i.e.*, from wild type poliovirus infections) resulted in poor signal-to-noise ratios.

Due to the lack of specificity we observed during isolations of viral RNP complexes from cells infected with PV1-3′MS2, we turned to alternate affinity tags that could increase the specificity of viral RNA isolations. In addition to adjusting affinity sequences from MS2 hairpins to aptamers, we also explored alternative genomic locations to the 3′NCR for insertion of these purification tags. The replication defect of polioviruses lacking the 3’NCR of the genome, including PV1-3′MS2, is thought to be a result of the absence of the complementary 5′ terminal region within the negative-sense intermediate RNA. This region, although not strictly required for viral replication, has been proposed to be a binding site for hnRNP C1/C2, allowing for efficient positive-sense RNA synthesis to take place [27]. Therefore, this recombinant virus was not ideal for identifying other proteins that may bind the 3′NCR of the poliovirus genome. Moreover, the coding region of the poliovirus genome is obviously restricted in its ability to tolerate exogenous nucleic acid insertions throughout its length due to the potential of altered viral protein production from disruptions to translation reading frame and/or irregular polyprotein processing. While there are examples of viable, recombinant polioviruses containing nucleic acid sequence insertions within the coding region of genomes to generate fusion or tagged viral proteins, these genomes are only quasi-stable [55,56]. To overcome the replication defect observed with PV1-3′MS2, the inherent deficiency this recombinant virus might offer in identifying proteins that interact with the genomic 3′NCR or negative-strand intermediate 5′ terminal region, and to promote the generation of a stable genome that could allow for growth kinetics that more closely matched wild type poliovirus, we focused on the 5′NCR to identify potential aptamer tag sequence insertion sites. While interference with regulatory RNA regions within the 5′NCR was a concern, there are several regions within the 5′NCR of the poliovirus genome that have previously been shown to tolerate sequence alterations with limited effects to viral replication processes. Nucleic acid sequences generated via systematic evolution of ligands by exponential enrichment (SELEX) to bind with high affinity to streptavidin (S1) or Sephadex (D8) were selected due to their short length and demonstrated potential for RNP complex isolation [42,43,44,45,46,47,48,49,50,51]. We tested three separate sites within the 5′NCR of the poliovirus genome: S-L III, S-L VI, and nucleotide position 702 for their capacity to tolerate aptamer insertions while maintaining biological activity. Making use of these regions, we generated poliovirus RNA constructs containing either of the two aptamer tags within the 5′NCR, in either a forward or reverse orientation to allow for aptamer sequences to be present in viral genomic RNAs or replication intermediate negative-strand RNAs and to permit strand-specific isolation from infected cells.

We initiated the RNP complex isolation study focused on the minimal, consensus sequences of the D8 and S1 aptamer tags with the rationale that their short length would be amenable to the production of recombinant virus containing a stable, RNA affinity-tagged genome. However, we observed insufficient RNA isolation efficiency associated with the S1 and D8 RNA affinity tags, and a loss of all specificity associated with these tags upon formaldehyde treatment. The minimal nature of these tags, particularly in the context of the relatively long poliovirus genomic RNA, likely limits the amount of affinity-tagged RNA that can be isolated. Our results agreed with the recent finding that the binding efficiency of the minimal S1 aptamer offers almost no specificity of isolation relative to an untagged RNA construct [34]. The authors of this latter study presented modified aptamer structures and tandem conformations that allow for up to 15-fold increases in binding efficiencies. However, the best binding efficiencies reported correspond to the inclusion of multiple tags of increased nucleotide length, likely preventing the generation of stable, recombinant poliovirus genomes containing these tags. In an attempt to increase the binding efficiency of the aptamer tags within the poliovirus 5′NCR, while maintaining the stability of the tags through multiple passages, we increased the stem length of both the D8 and S1 by 16 nucleotides to promote the accessibility of RNA affinity sequence to matrix. However, increasing the stem length of the aptamer tags also proved to be insufficient for isolating poliovirus RNP complexes from infected cells.

Aside from the identification of the 3’NCR and nucleotide position 702 of the poliovirus genome as amenable sites for the insertion of RNA affinity tags, the generation of recombinant viruses containing these tags, while not yet optimized for RNP complex isolation, revealed insights into the biology of the poliovirus genome. The Mahoney strain of the poliovirus 1 genome contains eight AUG triplets upstream of the authentic translation initiation site at nucleotide position 743, within the 5′NCR. Kuge and colleagues have previously demonstrated that insertions into the poliovirus genome that contain an AUG triplet resulted in base substitutions to the AUG triplet alone, with no other alterations to the insertion sequence [54]. Our results partially support this finding, in that the AUG triplet present in the S1 aptamer sequence was not tolerated in any of the viral isolates that were sequenced, with single nucleotide changes to this triplet sequence present in all cases. However, the sequence coding for the presence of the S1 aptamer within negative-sense RNA (S1−) also contains an AUG triplet (when present in the genomic RNA molecule) that is out-of-frame with authentic AUG start site. Interestingly, no alterations to protein production levels or polyprotein processing were observed upon *in vitro* translation of this construct compared to wild type RNA sequence. Whether this can be explained by the presence of a UAA stop codon in-frame with this AUG triplet remains to be seen. Other investigations have suggested that the translational efficiency of picornaviruses depends on the particular spacing between a polypyrimidine tract at nucleotide 558 of the 5′NCR and one of the cryptic AUG triplets at nucleotide position 586, as well as whether AUG triplets are present within structured or unstructured elements [57,58,59]. The function of the multiple AUG sequences upstream of the authentic translation initiation start site remains uncertain, but our work suggests that an additional AUG inserted into the poliovirus genome downstream of nucleotide position 586 is only selected against when this triplet is in-frame with the start codon, even when this AUG is within the structured region of an aptamer.

Our work also indicates that very short oligonucleotide sequences may not be competent for stringent isolation of the RNA when they occur within the context of a long, structured viral genome. Although the region between S-L VI and the translation start site of the poliovirus genome (including nucleotide position 702) is often considered more-or-less unstructured, recent global RNA structure analysis has suggested that this region of the genome may in fact contain higher-order structures [60]. Incorporating aptamer tags within a structured RNA will impact the native structure of that region, and may impact the way in which the inserted sequences are able to make intramolecular contacts, possibly affecting proper secondary structure formation of the tags themselves. It is also possible that RNA affinity tags incorporated into the 5′NCR of the poliovirus genome are sterically blocked from interacting with affinity matrices due to the highly structured nature of this region. Furthermore, the fact that this region interacts with many different protein species, which promote both viral translation and RNA replication, could lead to further masking of RNA affinity tags, thus blocking these RNA sequences from interacting with the matrix. MS2 coat protein RNA affinity tags within the 3′NCR of the poliovirus genome should be sufficiently separated from RNA structural elements within the genome, which may aid in the isolation of the tagged RNA, but our work suggests the MS2 RNA affinity purification scheme itself allows for untenable levels of non-specific protein co-isolation. Adding complexity is the fact that poliovirus RNA is at least partially duplexed during RNA replication, obstructing affinity matrix/tag interactions and further complicating isolation schemes.

The minimal S1 tag has recently been shown be highly inefficient in regard to its ability to retain RNA on the relevant affinity matrix, as untagged, negative-control RNAs are retained with similar efficiency [34]. Leppek and Stoecklin have demonstrated increased isolation efficiencies with the S1 aptamer by increasing both the stem length of this aptamer as well as the number of tags incorporated into the RNA of interest. Although it would no doubt be advantageous to incorporate multiple aptamer tags in series (and/or at the apex of a stem-loop structure) we were limited in our choice of sequence length and insertion site, due to the constraint that recombinant viral genomes containing RNA affinity tags must recapitulate the biological activities of the wild type poliovirus genome. The fact that there is a maximum size threshold for exogenous sequence insertions into the poliovirus genome before biological activity is affected was evidenced by cloning two modified streptavidin-binding aptamers into the nucleotide 702 region. This construct, S1m+(x2)702, produced the lowest levels of viral proteins of all constructs that were assayed by *in vitro* translation (Figure 11). Recent work has demonstrated the isolation of poliovirus RNA through infection of a cell line stably expressing uracil phosphoribosyltransferase followed by UV cross-linking and oligonucleotide-directed poly(A) isolation, suggesting that isolation of poliovirus RNA may be more tractable when using complementary oligonucleotide sequences rather than RNA affinity tags [61].

## 5. Conclusions

Overall, we have demonstrated that recombinant polioviruses containing RNA affinity tags within the noncoding regions of their genome are viable, and that these exogenous sequence insertions are stable for multiple passages in cell culture. Furthermore, we did not identify any significant compensatory mutations throughout the genomes of recombinant viruses as a result of these exogenous sequence insertions. Although we were able to specifically isolate RNA containing these affinity tags from cells infected with recombinant viruses, the efficiency of RNA affinity tag/matrix associations and isolation specificities were not sufficient to allow for the identification of novel cellular proteins that play roles in the RNA replication cycle of enteroviruses. However, the use of different affinity tags in the genomic positions discussed here could allow for this objective to be met in the future. Additionally, it is possible that these recombinant viruses have utility in other applications, such as in visualization of the production of specific polarities of RNA during the infectious cycle via the use of fluorescently labeled oligonucleotides complementary to tag sequences present in genomic RNAs or negative-strand intermediates [62]. In summary, the viruses and methods described here represent an initial step toward the isolation of RNP complexes associated with enterovirus RNA replication directly from infected cells, in a strand-specific manner. Investigations utilizing these tools could eventually aid in the identification of cellular proteins that could be specifically targeted by anti-enteroviral therapeutics.
